# Coupling human and natural systems for sustainable development

**DOI:** 10.1093/nsr/nwad086

**Published:** 2023-03-30

**Authors:** Bojie Fu

**Affiliations:** State Key Laboratory of Urban and Regional Ecology, Research Center for Eco-Environmental Sciences, Chinese Academy of Sciences, China

We are living in a time that many people refer to as the Anthropocene. In the Anthropocene, drastically changing landscapes and climates have resulted in social and environmental crises, such as ecosystem degradation, freshwater depletion and biodiversity loss. Coupled human and natural systems (CHANSs) are integrated systems in which people interact with natural components, which can provide theoretical insights for sustainable development in the Anthropocene. CHANSs are complex adaptive systems because of their ubiquitous feedback, full of non-linearity and chaos. With the progress of empirical, theoretical and modeling studies, the past two decades have witnessed a new phase of development in CHANSs, marked by a proliferation of frameworks for integrated research. An increase in the number of interdisciplinary programs has brought many integrated studies that successfully reveal new and complex patterns, processes, outcomes and feedback mechanisms from the holistic perspective of CHANSs.

The recent theoretical frameworks provide conceptual maps for rich CHANSs research, as well as the ability to respond to the issues raised by the theories and provide scientific support for sustainable development. Moreover, empirical research has enabled the analysis and justification of key concepts and processes in the framework at different scales using multiple sources of data and interdisciplinary approaches. These place-based approaches can take CHANSs research from theory to implementation.

This Special Topic highlights recent developments and crucial issues in CHANSs. It includes four Perspectives and three Review articles. The first, by Cumming, proposes a mechanistic framework for social–ecological mismatches, which helps to guide and direct our understanding of how transformational change, scale crossing actions and balancing endogenous and exogenous pressures can contribute to enhanced social–ecological resilience and sustainability [1]. The second, by Li *et al.*, discusses major challenges and opportunities for current CHANSs modelling and offers suggestions for future directions in which to advance science and its application, to promote the sustainability of CHANSs [2]. The third, by Pradhan, proposes three research avenues with a threefold scientific approach to address the urgent need to accelerate Sustainable Development Goal (SDG) progress and adopt a post-2030 agenda or a follow-up to the SDGs [3]. Cherubini *et al.* suggest that a sustainable transition in the agri-food sector requires a paradigm shift that embraces changes in lifestyles, agricultural practices and policies [4].

Bodin and Chen [5] review and synthesize recent network-centric studies, and use this network perspective to show how rangeland managers in a dynamic pastoral region in the Qinghai Province of China formed social relationships based on geographic proximity, social status and shared grazing areas. Liu [6] provides an overview of the metacoupling framework, highlights major advances in scientific discoveries and illustrates implications for global sustainability based on the existing applications of the framework, and also offers future perspectives with regard to filling important knowledge gaps and the need for tools and policy innovations. Fu *et al.* [7] propose a ‘landscape pattern—ecosystem service—sustainable development’ framework to describe China's landscape-scale ecological restoration and its impact on landscape patterns, ecological processes, ecosystem services for human well-being, sustainable livelihoods and socioeconomic development.

As a final note, I would like to thank all authors, editorial staff, international and domestic reviewers, and everyone else who has contributed to the initiation and completion of this Special Topic.

## Notes

Guest Editor of Special Topic and Editorial Board Member of NSR
